# Persistent Necrotizing Mediastinitis after Dental Extraction

**DOI:** 10.1155/2019/6468348

**Published:** 2019-11-07

**Authors:** Eugênia Leal de Figueiredo, Carolina Chaves Gama Aires, Bruno José Carvalho Macêdo Neres, Bruna Luna de Araújo, José Alcides Almeida de Arruda, Ricardo José de Holanda Vasconcellos

**Affiliations:** ^1^Department of Oral and Maxillofacial Surgery, School of Dentistry, Universidade de Pernambuco, Camaragibe, PE, Brazil; ^2^Department of Oral Surgery and Pathology, School of Dentistry, Universidade Federal de Minas Gerais, Belo Horizonte, MG, Brazil

## Abstract

Mediastinitis is a rare, progressive, and destructive infectious process due to cervical or odontogenic infections, which, if not diagnosed early, may lead to several complications, including airway involvement and even an imminent risk of death. Herein, we report an unusual case of a 37-year-old male with a bilateral submandibular hard swelling after the left third molar extraction. After surgical intervention with submandibular drainage and antibiotic therapy, the infection persisted without explanation, since the patient was not hypertensive, did not have diabetes mellitus or sexually transmitted infections such as HIV or syphilis, and did not smoke or drink alcoholic beverages. A thoracic surgeon then intervened, treating the mediastinitis surgically by drainage, thus obtaining a significant improvement of the patient's health. Mediastinitis is a serious condition. Clinicians and maxillofacial surgeons should be alert to make an immediate diagnosis and select the appropriate treatment in order to prevent worsening of the patient's clinical condition.

## 1. Introduction

Odontogenic infections are among the most common conditions affecting the oral and maxillofacial region and can lead to severe complications, including airway obstruction, such as Ludwig's angina, septic thrombosis of the cavernous sinus, necrotizing fasciitis, and mediastinitis [1, 2]. They are usually limited to the etiology of origin; however, under certain circumstances involving vascular obliteration with microthrombosis around the locus of infection, accompanied by acute inflammation of subcutaneous tissue and swelling of the underlying tissues with progression of the condition, there is no local intravascular coagulation and the infected tissue becomes necrotic. Thus, the infectious process may spread and overcome bone, muscle, and mucosal barriers, coming into contact with adjacent facial spaces and resulting in severe infections in cavities of the body [2].

Mediastinitis is a rare, progressive, and destructive disease that may often lead to an imminent risk of death [3]. It originates from an infection in a deep cervical space. Sixty to 70% of reported cases are due to odontogenic infections, especially those involving extractions of mandibular third molars [3, 4]. Epidemiological data estimate a mortality rate of 4.8 per 1,000,000 per year, with higher incidence rates among African, Hispanic, and American-Indian populations [4]. In contrast, a lower incidence has been documented among Asians [4]. Due to the robust blood supply, infection in the head and neck region is uncommon, accounting for 1% to 10% of cases [5].

Mediastinitis has a fulminating course with a potential risk of sepsis, pericarditis, and multiple organ failure. The main reasons cited as crucial factors in the poor prognosis of the disease are a late diagnosis and inappropriate surgical approaches. The complications resulting from odontogenic infections are uncommon, and therefore, it is important to use a well-directed anamnesis and to choose responsibly the appropriate treatment for these infections [6]. Immediate diagnoses with aggressive surgical treatment and careful surveillance may limit the progression of the disease [7, 8]. Thus, the aim of the present report was to present an unusual case of a young adult with persistent necrotizing mediastinitis after dental extraction. This article provides information that could help clinicians and maxillofacial surgeons with the diagnosis and management of this condition.

## 2. Case Presentation

**Figure 1:**
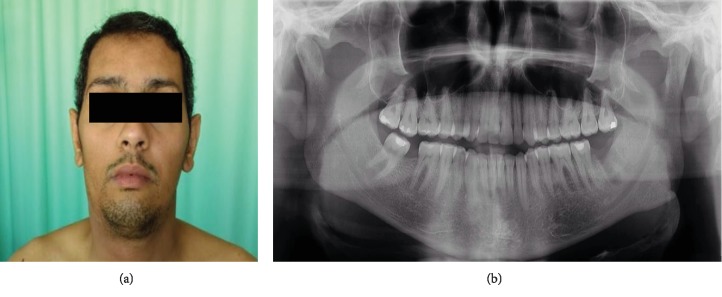
(a) Initial clinical aspect showing swelling in the submandibular region. (b) Panoramic radiograph taken after removal of the left mandibular third molar.

A 37-year-old male patient presented at the emergency service of oral and maxillofacial surgery of a private hospital in Recife, Brazil, with complaints of a painful hard swelling in the submandibular region (Figure 1(a)). He had undergone extraction of the left third molar seven days before presentation (Figure 1(b)). He has noncontributory medical or socioeconomic history, since he was not hypertensive, did not have diabetes mellitus or sexually transmitted infections such as HIV or syphilis, and did not smoke or drink alcoholic beverages.

**Figure 2:**
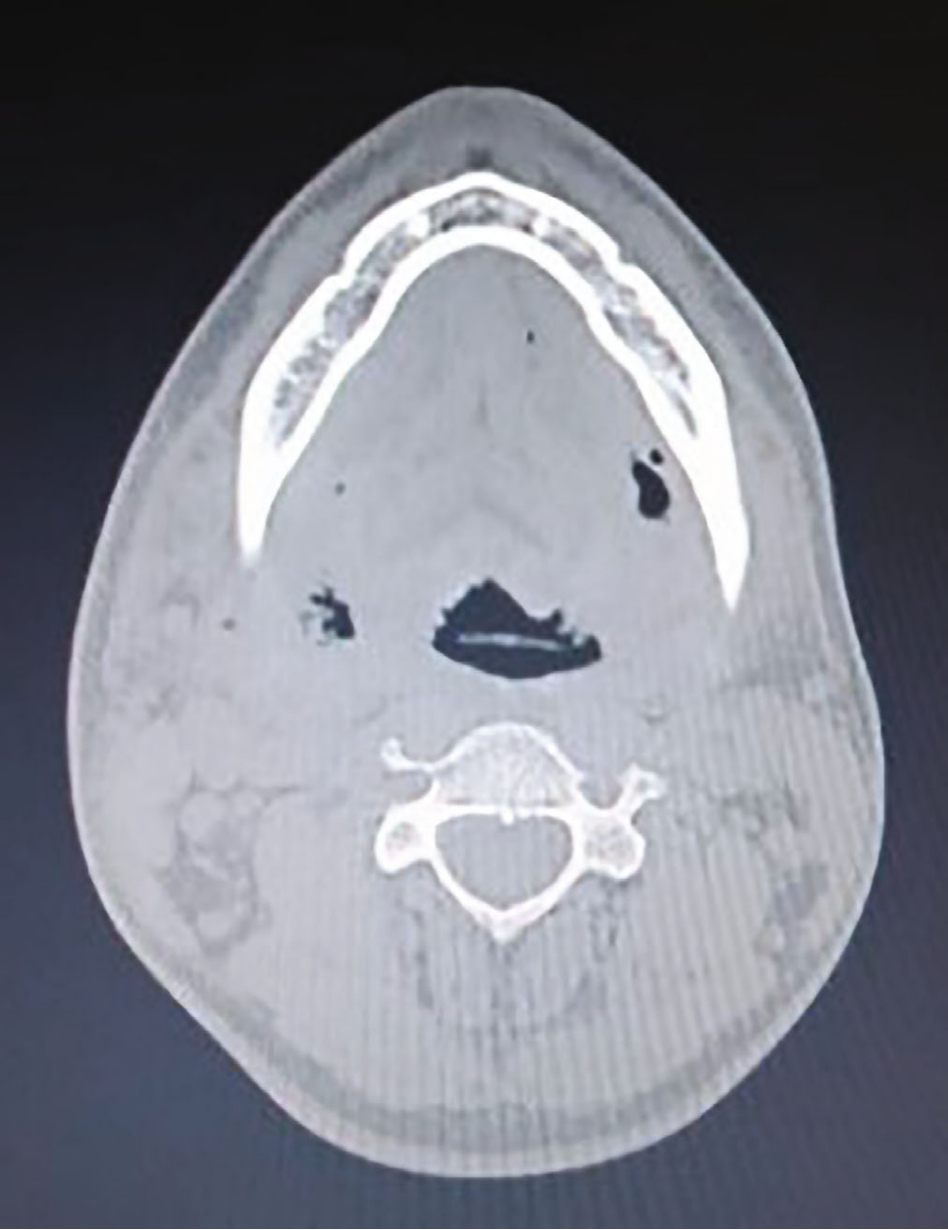
Computed tomography axial view showing the initial aspect of the patient's face.

A computed tomography revealed a hypodense image in the submandibular region accompanied by the presence of gas in their interior (Figure 2). Respiratory rate and blood pressure changes were not observed. On extraoral examination, the skin of the submandibular region was tender bilaterally and of normal color. Intraoral examination revealed maximal interincisal opening (<20 mm), as well as absence of the left mandibular third molar. Blood cultures were performed, and intravenous Metronidazole (500 mg, 8/8 hours) and Rocefin (1 g, 12/12 hours) were empirically started.

On the third day, there was a slight hyperemia in the cervical region and in the thorax, and the patient reported dysphagia and dysphonia. Due to the persistence of the infection, the antibiotic was changed and a new cycle was started with 1.5 g Tazocin injected intravenously every 6 hours. To monitor the clinical status of the patient, daily laboratory tests were carried out, which showed that the body continued to exhibit marked leukocytosis (15,290). There was also positivity and an increase in the 7.32 reference value for C-reactive protein, indicating that there was still a significant infection in the body.

**Figure 3:**
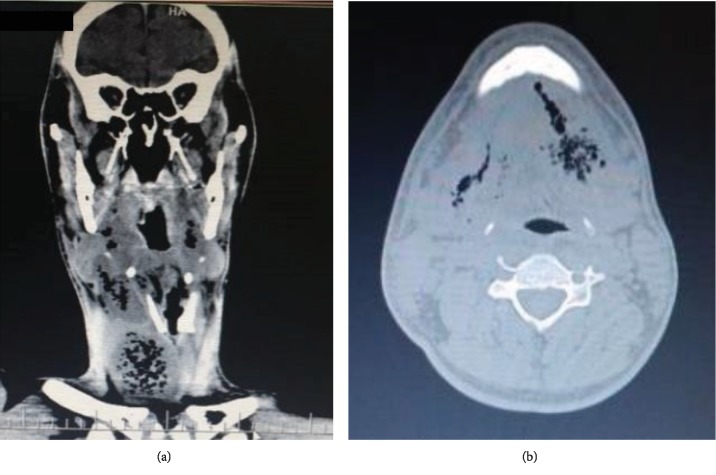
(a, b) Computed tomography of the face and cervical region revealing soft tissue enlargement associated with subcutaneous emphysema between muscular planes. Purulent mediastinal collections with gaseous contents and tracheal deviation can be seen.

**Figure 4:**
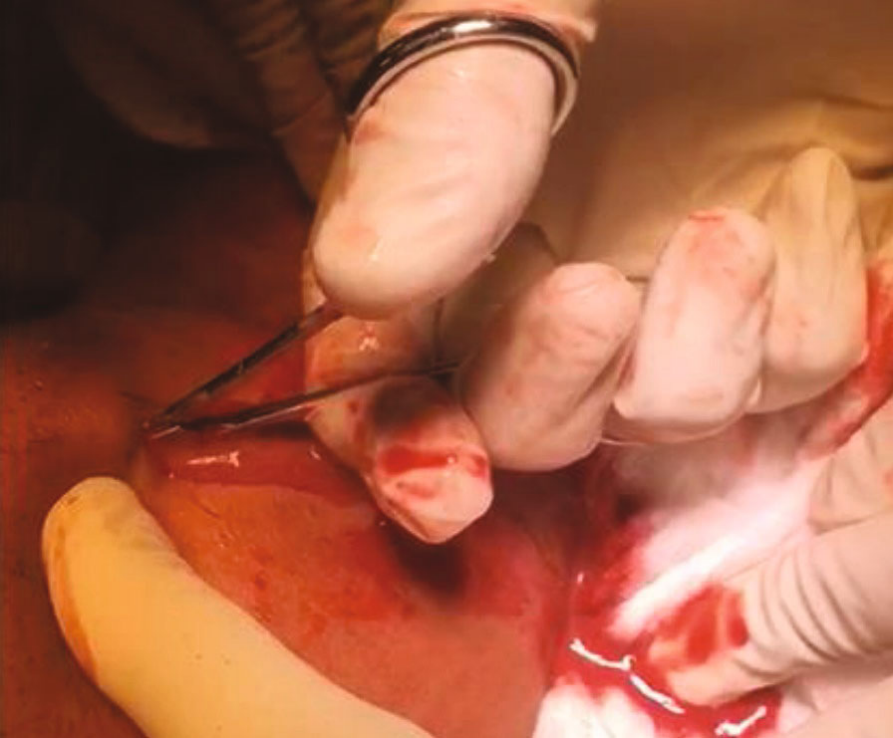
Submandibular incision and purulent collection drainage.

A new contrast-computed tomography of the face, neck, and chest clearly showed the presence of a purulent collection in pharyngeal spaces, with a descending path to the mediastinum and marked deviation of the trachea (Figure 3). In view of the emergency situation, after antisepsis of the region, bilateral drainage of the submandibular and submental regions, and tissue divulsion, irrigation with copious amounts of 0.9% saline solution to serve as drainage orifices was performed on the 5th day of hospitalization (Figure 4). Next, two No. 2 Penrose drains were installed in drainage holes, and the patient was also referred to the intensive care unit (ICU) for 5 days due to the severity of his condition and the need for daily care. The patient underwent orotracheal intubation and, because of severe limitation of mouth opening, the aid of a bougie, an artifact used for intubation of a difficult airway, was selected. During the intensive care period, an investigation was carried out in search of data that could explain the poor evolution of the patient, since he had no comorbidities. A chest tomography revealed the presence of purulent collection in the region above the sternal furcula.

Due to a clinical presentation without significant improvement, the thoracic surgery team intervened six days after the procedure performed by the maxillofacial surgeons. The decision was made to perform tracheostomy and suprasternal cervicotomy, which revealed the presence of necrotic tissues mainly consisting of omohyoid musculature. A large pseudocyst extending from the submandibular region to the sternal furcula and containing secretion was also detected. A tracheal aspiration culture was performed and revealed *Pseudomonas aeruginosa* and *Klebsiella pneumoniae ssp pneumoniae*. Opening of the mediastinum revealed no devitalized tissues and very few purulent collections. A Penrose drain was installed in the furcula, and at the end of the procedure, the patient underwent a tracheostomy and returned to the ICU.

During the postoperative period, the patient was kept on antibiotic therapy with 1 g Meropenem 8/8 hours and 400 mg Targocid 12/12 hours intravenously, showing significant improvement with sudden falls in leukocytosis and C-reactive protein, as well as improvement of general clinical status. After 1 year of treatment, the patient was invited by telephone to follow-up; however, he did not return to our service and only reported no symptomatology.

## 3. Discussion

Mediastinitis is defined as inflammation of mediastinal tissues and is often characterized by rapidly progressive bacterial infection on multiple tissue planes, leading to vascular, muscular, and airway involvement. In the present case, the infection originated due to tooth extraction. The infectious process reached submandibular, cervical, and mediastinal regions, in agreement with Fukuchi et al.'s explanation [9], i.e., odontogenic infections of the oropharynx can expand to the mediastinum through respiratory movements and negative intrathoracic pressure.

The literature suggests a higher prevalence of males in the development of deep cervical infections, possibly due to less care with oral hygiene [6]. According to Qu et al. [3], submandibular and pterygomandibular spaces were those most involved in the disease. This fact can be explained by anatomical reasons, since 75% of the odontogenic infections were due to extractions of mandibular molars, which eventually drain bacteria into these spaces. This is what happened to the present patient, with the infection evidently involving the submandibular space bilaterally.

Regarding the radiographic characteristics, the image is a diffuse and often well-defined area, demonstrating the increase in volume of the affected region. In the lateral radiograph of the neck, it is common to observe a displacement of the posterior wall of the pharynx with the presence of gas. When the infection progresses towards the mediastinum and the thorax, as observed in the anteroposterior radiograph of the thorax, an increase of the mediastinal space occurs, with air accumulating in this region [10].

Among the main signs and symptoms, the presence of dysphagia, pain, and edema in the cervical region, chest pain, and airway involvement should draw attention [3]. In addition to all the signs and symptoms mentioned, the present patient had a necrotic tissue region, which explains the severity of his infection. Furthermore, elevated levels of C-reactive protein, i.e., concentrations between 1.47 and 7.29, have indicated the need for surgical intervention in a number of cases of documented severe odontogenic infections [10–12]. This also applied to the current patient. Thus, the maxillofacial surgeon should always request laboratory tests, analyzing the number of leukocytes and C-reactive protein levels, since high values show that the patient has an odontogenic infection.

Regarding patient management, Martins et al. [11] showed that penicillin is the most used and therefore the first-choice drug, which may be associated with *β*-lactamase inhibitors if necessary. However, in the event of failure or allergy, clindamycin and the macrolide class of drugs, particularly azithromycin, are viable alternatives, along with moxifloxacin, which has shown promising results in clinical trials. Here, the antibiotic therapy used was Metronidazole and Rocefin at the beginning, with a later switch to Tazocin and then to Meropenem and Targocid after drainage.

Considering the severity and rapid spread of the infection in the present case, it became clear that the maxillofacial surgeon should not underestimate its occurrence and should always be prepared for the appearance of deep infections, analyzing the case quickly for diagnosis and treatment and thus protecting the life of the patient. Therefore, in the presence of a severe odontogenic infection, we emphasize the importance of a multidisciplinary team consisting of a thoracic surgeon, general practitioner, and infectologist for better handling of the case and greater chances of successful treatment.

In summary, early diagnosis, a surgical approach involving drainage, and adequate antibiotic therapy are three key factors in the successful treatment of mediastinitis, a condition with a rapid and destructive course which can lead to death.

## References

[1] Prado-Calleros H. M., Jiménez-Fuentes E., Jiménez-Escobar I. (2016). Descending necrotizing mediastinitis: systematic review on its treatment in the last 6 years, 75 years after its description. *Head Neck*.

[2] Arruda J. A., Figueiredo E., Álvares P. (2016). Cervical necrotizing fasciitis caused by dental extraction. *Case Reports in Dentistry*.

[3] Qu L., Liang X., Jiang B., Qian W., Zhang W., Cai X. (2018). Risk factors affecting the prognosis of descending necrotizing mediastinitis from odontogenic infection. *Journal of Oral and Maxillofacial Surgery*.

[4] Gunaratne D. A., Tseros E. A., Hasan Z. (2018). Cervical necrotizing fasciitis: systematic review and analysis of 1235 reported cases from the literature. *Head Neck*.

[5] Hidaka H., Ozawa D., Kuriyama S. (2017). Risk factors for delayed oral dietary intake in patients with deep neck infections including descending necrotizing mediastinitis. *European Archives of Oto-Rhino-Laryngology*.

[6] Adoviča A., Veidere L., Ronis M., Sumeraga G. (2017). Deep neck infections: review of 263 cases. *Otolaryngologia Polska*.

[7] Sarna T., Sengupta T., Miloro M., Kolokythas A. (2012). Cervical necrotizing fasciitis with descending mediastinitis: literature review and case report. *Journal of Oral and Maxillofacial Surgery*.

[8] Gore M. R. (2018). Odontogenic necrotizing fasciitis: a systematic review of the literature. *BMC Ear, Nose and Throat Disorders*.

[9] Fukuchi M., Suzuki O., Nasu D. (2015). Descending necrotizing mediastinitis treated with tooth extractions following mediastinal and cervical drainage. *Case Reports in Gastroenterology*.

[10] Jiménez Y., Bagán J. V., Murillo J., Poveda R. (2004). Odontogenic infections. Complications. Systemic manifestations. *Medicina oral, patología oral y cirugía bucal*.

[11] Martins J. R., Chagas O. L., Velasques B. D., Bobrowski Â. N., Correa M. B., Torriani M. A. (2017). The use of antibiotics in odontogenic infections: what is the best choice? A systematic review. *Journal of Oral and Maxillofacial Surgery*.

[12] Ylijoki S., Suuronen R., Jousimies-Somer H., Meurman J. H., Lindqvist C. (2001). Differences between patients with or without the need for intensive care due to severe odontogenic infections. *Journal of Oral and Maxillofacial Surgery*.

